# Asgard archaea do not close the debate about the universal tree of life topology

**DOI:** 10.1371/journal.pgen.1007215

**Published:** 2018-03-29

**Authors:** Violette Da Cunha, Morgan Gaia, Arshan Nasir, Patrick Forterre

**Affiliations:** 1 Institut Pasteur, Unité de Biologie Moléculaire du Gène chez les Extrêmophiles (BMGE), Département de Microbiologie, Paris, France; 2 Institute for Integrative Biology of the Cell (I2BC), CEA, CNRS, Univ. Paris-Sud, Université Paris-Saclay, Gif-sur-Yvette cedex, France; 3 Department of Biosciences, COMSATS Institute of Information Technology, Islamabad, Pakistan; Vanderbilt University, UNITED STATES

In Da Cunha et al. [1], we provided evidence that the results by Spang et al. [2] positioning the Lokiarchaea as the link between prokaryotes and eukaryotes were deeply influenced by the inclusion of likely contaminated sequences, the presence of fast-evolving species (FES), and the choice of phylogenetic markers. Our analyses revealed the presence of two phylogenetic signals within the concatenated universal markers: one supporting a two domains (2D) scenario, and the other supporting a three domains (3D) scenario. Our extensive RNA polymerase large subunits phylogenetic analyses strongly supported the latter [1]. Far from disregarding the other Asgards [3,4], Thorarchaea sequences were extensively used for reanalysing the universal markers, and most Asgard sequences were included in our RNA polymerase analyses. Altogether, our results suggested that the Asgards are not the ancestors of Eukarya but a sister group to Euryarchaeota.

## There is evidence of contamination in the lokiarchaeal genomes

On top of the very high heterogeneity detected in Loki’s genome (78.21%), our quality analyses with CheckM (Parks, Skennerton, Imelfort, http://ecogenomics.github.io/CheckM/)) and Anvi’o (Meren, http://merenlab.org/software/anvio/) also revealed a high contamination index (between 45% and 57%), necessarily underestimated because quality analyses are limited to defined sets of markers [5]. Considering a good part of this contamination index as the result of the heterogeneity still leaves room for actual contamination from other sources. There is no need for important contamination to bias phylogenetic reconstruction if it is located in strong markers, as we have shown with the elongation factor 2 (EF2) [1].

**Fig 1:**
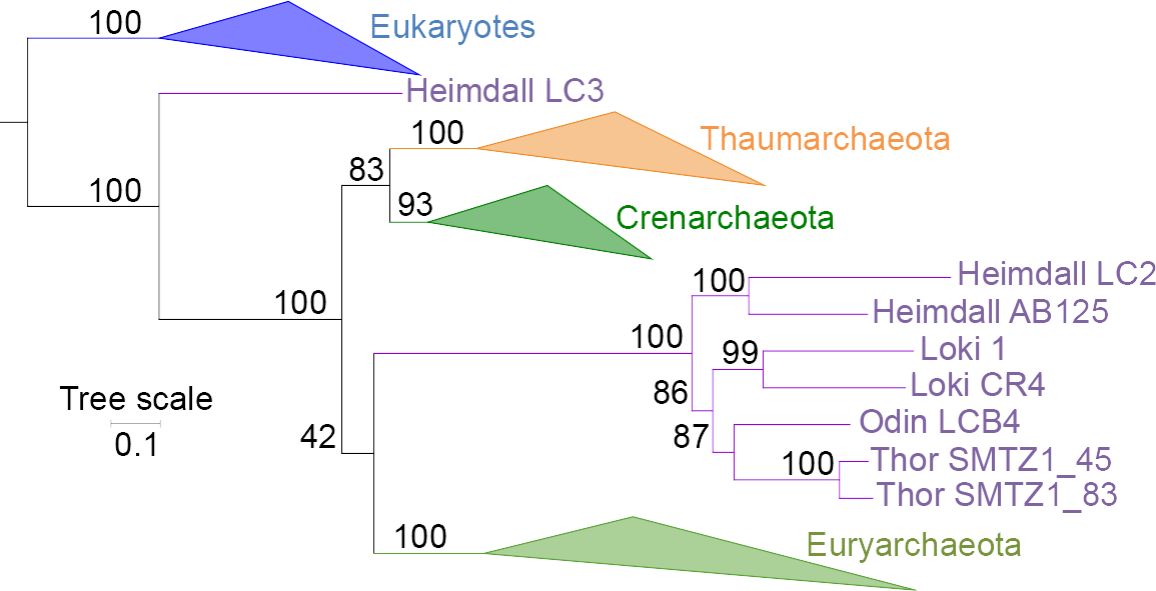
Maximum-likelihood tree of EF2 without bacteria. Maximum-likelihood single-protein tree of EF2 without bacteria (LG+R6 model) after inclusion of Asgard sequences. Eukaryotes, Thaumarchaeota, Crenarchaeota, and Euryarchaeota are indicated in blue, orange, dark green, and light green, respectively. Asgard sequences are indicated in purple. Odin and Thorarchaea seem to possess probable paralogs retrievable with BLASTp searches that were not included because they are different in length than the other Asgard sequences and contain specific indels. The scale bar represents the average number of substitutions per site. Values at nodes represent support calculated by nonparametric bootstrap (out of 100).

We could identify such a likely contamination in EF2 by detecting long insertions matching to eukaryotic paralogs, essentially in Heimdall LC3 (formerly Loki 3) [1]. No other Asgard sequences [3] contain the LC3-specific insertions, reinforcing the contamination hypothesis. In an EF2 single-protein tree without bacteria to increase the signal, Heimdallarchaeota are not monophyletic, with LC3 still branching with eukaryotes, whereas all the other Asgards are sister groups to Euryarchaeota (Fig 1), suggesting that patches of contaminating sequences indeed remained in Heimdall LC3 after trimming. The complex evolutionary history of this protein could perhaps explain the accumulation of artificial insertions in the LC3 genome. We suspected that additional hidden patches of contamination could similarly be present elsewhere in other Asgard universal proteins, especially in Heimdall LC2 and LC3 reconstructed through a Multiple Displacement Amplification (MDA) process. This could explain why not only Asgards belonging to different phyla (Lokiarchaea, and Heimdallarchaea [represented by the formerly named Loki2 and Loki3]) but also to the same phylum (Heimdallarchaeota) were not always monophyletic in our single-protein analyses [1].

We suspected that patches of contamination could also be present in Heimdall LC3 RpoA since this subunit is encoded by a single gene in LC3 (like in Thaumarchaeota and the related Bathyarchaea and Aigarchaea), whereas all other Asgards have a dimeric version (like all Crenarchaeota, all Euryarchaeota, and most DPANN). Unlike Spang et al. [4], we never observed best hits to Lokiarchaea nor to the two other Heimdallarchaeotes using the complete LC3 sequence (best hits to Bathyarchaea only) or various portions of N- and C-terminal sequences (Loki3/Heimdall LC3 RpoA access numbers: AKC94880/OLS19521). In contrast, we recovered best hits to other Asgards when using Heimdallarchaeote LC2 or AB_125 sequences. The branching of LC3 at the base of Asgards in the RpoA tree [3] could indicate that LC3 RpoA contains Heimdallarchaeote sequences mixed with sequences from other archaea related to Bathyarchaea.

## The presence of FES in dataset favours 2D topologies

Spang et al. [4] argued that their 2D trees are not affected by Long-Branch Attraction (LBA), because LBA would have attracted eukaryotes outside the Archaea. However, the branching of Archaea in the well-known fast-evolving *Methanopyrus kandleri* in the 2D tree of Spang et al. [2] clearly reveals an LBA artefact. The Euryarchaea could then be possibly attracted outside Archaea because of their evolutionary relationship with DPANN [6–8]. This probably explains why removing FES dramatically increased (from 1 to 11) the number of trees recovering the 3D topology [1].

## There is no strong LBA affecting the position of Eukarya in the 3D trees

**Fig 2:**
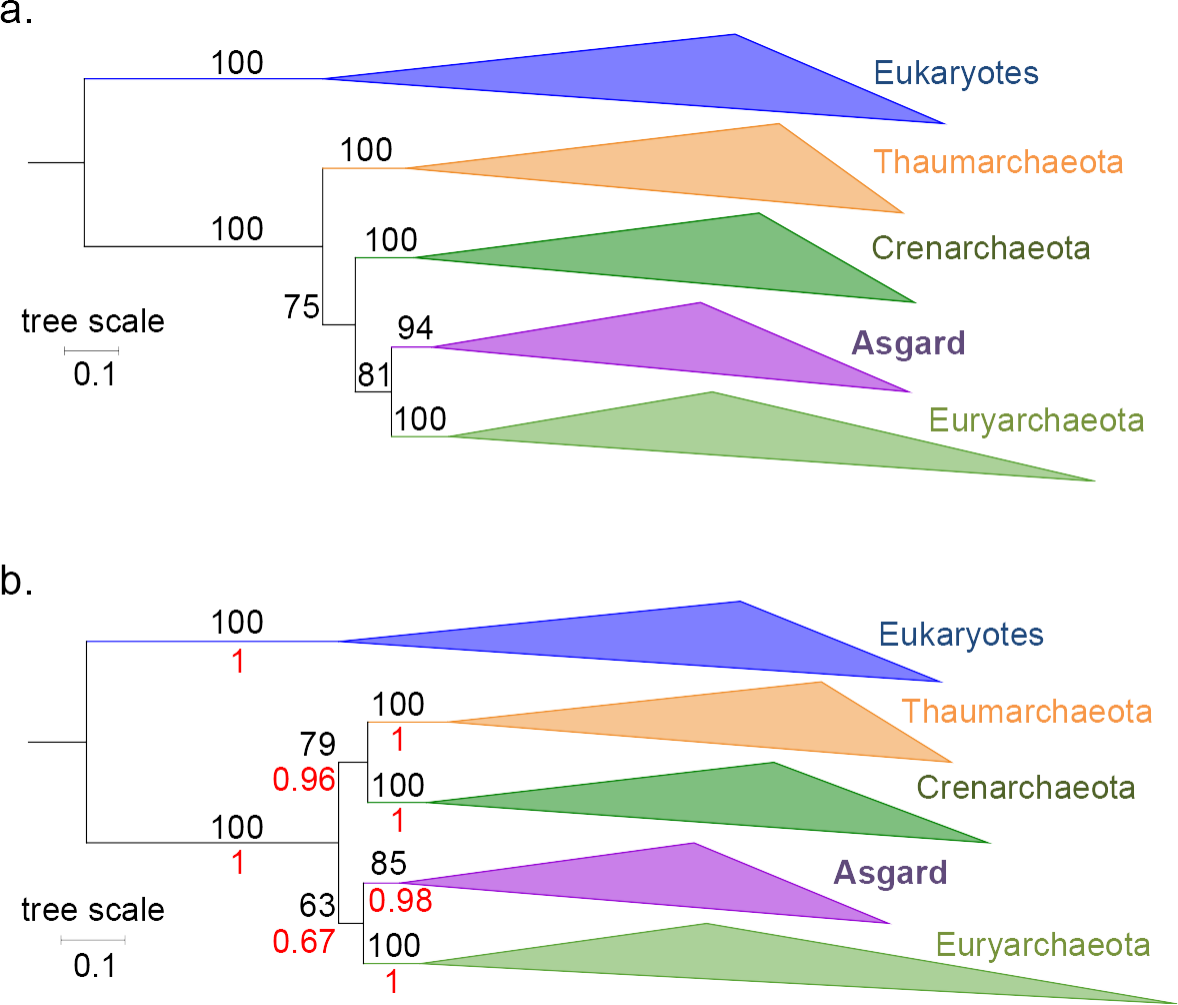
Phylogenetic trees of the concatenations of the 11 Woese proteins and the 6 AU-relevant Woese proteins, without bacteria. **(a)** Maximum-likelihood tree of the concatenated 11 Woese proteins with the Asgard sequences (LG+R7). No satisfactory convergence could be obtained in Bayesian inference with the CAT-GTR model at the time of this submission. **(b)** Phylogenetic tree of the concatenated 6 AU-relevant Woese proteins with the Asgard sequences. ML (LG+F+R8) and Bayesian inference (CAT-GTR model; maxdiff = 0.09) trees were identical, and supports from both were reported on the topology (nonparametric bootstrap, out of 100, in black, and posterior probabilities in red, respectively). All other combinations of chains (4 independent chains)—despite moderate convergences (maxdiff between 0.01 and 0.03)—yielded the same global topology, with the Asgards grouped with Euryarchaeota. For both trees, the scale bar represents the average number of substitutions per site.

Spang et al. [4] argued that LBAs were affecting the 3D trees we obtained (i.e., with the concatenated 6 AU-relevant Woese and the 11 Woese proteins, and the RNA polymerase [1]), resulting in the misplacement of Eukaryotes between the bacterial outgroup and the Archaea. This seems unlikely for the RNA polymerase, since we have shown that Asgards remained a sister group to Euryarchaeota (Archaea rooted in Thaumarchaea) in the absence of bacteria (Figure S35 in [1]). We have now performed similar analyses with the two concatenated Woese protein datasets after inclusion of all available Asgard sequences and obtained trees again displaying the grouping of Asgards with Euryarchaeota (Fig 2). These results strongly support the absence of strong LBA affecting the position of Eukarya in the 3D trees. If an LBA artefact was indeed misplacing the Eukaryotes, removing the bacteria would have dramatically impacted the trees. Our new analyses corroborate instead the position of the putative Asgard superphylum as a sister group to Euryarchaeota.

## Phylogenetic analyses excluding EF2 are compatible with the presence of conflicting signals within the universal markers

We never claimed that the Asgard/Eukarya affiliation could not be obtained without EF2. We ourselves obtained it when we removed EF2 from the concatenation of the eocyte proteins (Figures S26, S27 in [1]). Zaremba-Niedzwiedzka et al. indeed obtained significant support in a maximum-likelihood framework for a 2D universal proteins tree based on 48 universal markers without EF2 [3]. However, their species dataset still contained many FES that could introduce a bias favouring 2D trees [1]. Furthermore, they could not obtain any “good” (maxdiff <0.1) nor “acceptable” (maxdiff <0.3) convergence in Bayesian framework with the CAT-GTR model (Supplementary Table 4 in [3]; see Phylobayes manual) and hence could not corroborate their results with this approach. Consequently, despite having more Asgard taxa, Zaremba-Niedzwiedzka et al. [3] could not obtain results as congruent and robust as those obtained by Spang et al. [2] once they removed EF2. The lack of convergence in their Bayesian analysis is actually compatible with the presence of conflicting signals within the universal proteins, previously hidden by the presence of EF2 [1].

Finally, the confirmed presence of many Eukaryotic Signature Proteins (ESPs) in the genomes of the additional Asgards [3] cannot be seen as a confirmation of their grouping with Eukarya since their presence could also be explained by their losses in other archaeal lineages, as suggested for ESPs in Thaumarchaeota [9–11], or ancient gene transfers with proto-eukaryotes.

## Conclusions

Spang et al. [4] stated that we 1) used “inadequate methodology”, without explaining, 2) “misinterpret[ed] data” while presenting some of our findings out of their context, and 3) “ignore[d] previous work”, which our publication is actually based on. Results, criticism, and debates should be welcome in science and not trigger hostility. Although our analyses presently favour the 3D topology, we consider that the relationship between Archaea and Eukarya is still an open question requiring more studies.
